# Editorial: Psychology for the common good: The interdependence of citizenship, justice, and well-being across the globe

**DOI:** 10.3389/fpsyg.2022.934456

**Published:** 2022-09-26

**Authors:** Isaac Prilleltensky, Salvatore Di Martino, Ottar Ness

**Affiliations:** ^1^Department of Educational and Psychological Studies, University of Miami, Miami, FL, United States; ^2^Department of Psychology, University of Bradford, Bradford, United Kingdom; ^3^Department of Education and Lifelong Learning, Norwegian University of Science and Technology, Trondheim, Norway

**Keywords:** social justice, citizenship, wellbeing, communities, mattering, inequality, equity, human rights

## Introduction

Imagine a society where people live in communities where everyone feels valued and adds value to others. Furthermore, imagine that everyone benefits from relationships, policies, and practices characterized by fairness and justice. In such society, it is very likely that people will experience also high levels of wellness. Prilleltensky (2012, p. 2) has defined wellness as “a positive state of affairs, brought about by the simultaneous and balanced satisfaction of diverse objective and subjective needs of individuals, relationships, organizations, and communities.” These needs depend on the fulfillment of *personal* (empowerment, sense of control, competency, and resilience), *relational* (empathy, compassion, inclusion, social support, social capital, and psychosocial accompaniment), and *communal* factors (social justice, fairness, equity, and equality) (Prilleltensky, 2001; Watkins, 2019; Riemer et al., 2020; Di Martino et al., 2022). However, in order to achieve a state of wellness, fairness, and worthiness, everyone in the community must actively pursue the common good. This is why we need citizenship, understood in Aristotelian terms as an active form of participation in political life (see Morrison, 1999). The challenge is how the public sector, citizens, and stakeholders can work together to support the interdependence of citizenship, social justice, and wellbeing across the globe so that no one in society is left behind (United Nations, 2015; United Nations Department of Economic Social Affairs, 2016). We aim to contribute to that goal by bringing together a collection of articles showing philosophical principles, empirical links, and interventions that promote the interdependence of citizenship, justice, and wellbeing.

What we know already is that citizenship is affected by, and, in turn, affects justice and wellbeing (Prilleltensky, 2012; Quinn et al., 2020). However, there is a need to understand how citizens create conditions that support both justice and wellbeing, at the micro, meso, and macro levels of analysis (Di Martino et al., 2022). Following Di Martino et al. (2022, p. 19), there is a need to develop “a psychosocial theory of the common good, which connects fairness with social, economic, cultural, and political factors that are related to national wellbeing.”

Although there is emerging research showing that social justice and citizenship are predictive of individual, relational, community, and national wellbeing (Di Martino and Prilleltensky, 2020; Di Martino et al., 2022), more research is required to explore the interdependence among these constructs across geographical locations and settings as well as across diverse populations. There is also a need for work that investigates how these constructs are predictive of individual and collective outcomes in fields as diverse as health, education, economic output, autonomy, and solidarity. For example, the literature tells us that both social justice and citizenship are closely associated with a wide range of important individual and societal wellbeing outcomes like social connectedness and integration, personal and collective mattering, innovation, productivity, work and school performance, improved health, trust, healthy behavior, and longevity (Marmot, 2015; Holmberg and Rothstein, 2017; Wilkinson and Pickett, 2018; Word Health Organization, 2019; Marmot et al., 2020). However, there is a paucity of research that integrates these findings. The aim of the current Research Topic is to explore the interdependence among social justice, citizenship, and wellbeing across diverse communities and multiple levels of analysis, toward a psychology of the common good.

Taken together, the articles in this Research Topic demonstrate the central role of mattering in citizenship and wellbeing. We define mattering as experiences of feeling valued and adding value (Prilleltensky and Prilleltensky, 2021). To feel like they matter, people need to feel valued by self and others, and they need to contribute to self and others, including family members, friends, colleagues, and citizens in the community. As shown in many of the papers in this issue, people thrive when they feel recognized, appreciated, and treated with dignity, and when they have an opportunity to participate in community life. The vital role of mattering in citizenship, justice, and wellbeing, is seen in three strands of articles. First, we notice the importance of mattering in psychosocial interventions. Second, we observe the roles of feeling valued and adding value in empirical investigations linking various aspects of citizenship with justice and wellbeing. Finally, we identify mattering as a unifying concept in philosophical and cultural approaches to the common good. We explore these three sets of paper below.

## The role of mattering in psychosocial interventions linking justice, citizenship, and wellbeing

A clear goal in many of the interventions described in this Special Research Topic is to make people feel valued and to offer them meaningful opportunities to add value, either to themselves or to the community around them. While some papers discuss interventions that operate at more than one level, in general the papers address program and policies that function at the micro, meso, and macro levels. In all cases, they talk about the importance of granting recipients of services or activists a sense of mattering. There are many ways to help people build or restore a sense of mattering in their lives. In some cases, it is as simple as giving them a sense of control over their lives and their ability to care for a pet in their supported living facilities (see Friesinger et al.). In others, it is a matter of creating inclusive practices where people with mental health challenges feel included and participate in their treatment plans (see Brekke et al.; Sundet; Smith et al.; Gabhann and Dunne in this issue).

There are multiple ways in which these interventions promote respect, dignity, and inclusion. In some cases, it has to do with listening to the voices of people with mental illness; in others, it involves co-creating an action plan with professionals and family members. In all instances, the interventions described in these papers demonstrate the positive effects of enabling participation of clients in decision making about their living conditions, supports needed, or course of treatment. In every situation, the wellbeing of people with psychosocial challenges is improved by either making them feel valued or ensuring that they add value to themselves or others. Asking for their opinions conveys a sense of respect. Supporting them emotionally makes them feel loved. Enabling them to add value gives them a sense of control, competence, mastery, autonomy, and self-efficacy (Prilleltensky and Prilleltensky, 2021). These are the virtues of co-creation in mental health care.

A second set of interventions target meso level settings such as mutual aid groups (Fernandez-Jesus et al.), social action for climate change (Ursin et al.); or school programs to contribute to the wellbeing of the community (Sepulveda et al.). The authors describing these interventions clearly show the importance of contributing to something or someone beyond the self. Participants in these programs helped other people with COVID-19, engaged in social action to stop climate change, and work with other students in school to add value to their communities. Sepulveda et al. investigate the efficacy of a school-based program called MPOWER, which is a program designed to help adolescents engage in actions that benefit the community. As the authors report, “the MPOWER program was associated with an increase in the BTS (Beyond the Self) aspect of purpose and self-efficacy among participants. Especially promising are findings that suggest that participants' internal attitudes about their own abilities to positively impact their world and susceptibility to external pressures can be changed through participation in MPOWER.”

In this set of interventions participants added value to diverse groups of people and settings, but they all contributed to the common good. These citizen-initiated projects gave people meaning and purpose, which are essential components of wellbeing.

A third set of papers address systemic interventions dealing with punishment in schools and society (Gaete-Silva and Gaete), community-wide interventions (Montiel et al.), and social policies (Krokstad). These papers demonstrate the crucial role of justice and fairness in promoting wellbeing. Furthermore, they argue that wellness cannot be fully achieved in the absence of fairness. Montiel et al. for example, identify the limitations of positive psychology interventions that focus almost exclusively on intrapsychic efforts, rendering social conditions invisible. Krokstad, in turn, makes the case for creating conditions of fairness where all citizens can matter.

In total, 11 papers in the Research Topic discuss the powerful role of mattering in citizenship and wellbeing. Some of these papers also argue that fairness is a prerequisite for mattering, which is, in turn, a sine qua non condition for wellness. In the next section, we explore the empirical links among mattering, citizenship, justice, and wellbeing. These constructs constitute building blocks for the common good.

## The role of mattering in empirical investigations linking justice, citizenship, and wellbeing

Seven papers in this Research Topic explore various aspects of citizenship and the common good. In all of them we see a distinct role for one or more aspects of mattering in fostering wellbeing and the common good. Scarpa et al. using Co-variance Based Structural Equation Modeling, demonstrated a direct effect of mattering onto wellbeing and an indirect effect of justice onto wellness through mattering. Their findings demonstrate that mattering is likely to fully mediate the relationship between fairness and wellness in multiple domains of life, such as psychological, interpersonal, physical, communal, and occupational wellbeing. Their study shows that conditions of fairness lead to experiences of worthiness, which, in turn, contribute to wellness.

The ability to add value or make a contribution to society is central to mattering. Perkins et al. show in an international comparative study that grassroots activism, civil liberties and political rights, decentralization, and voter participation correlate with the United Nations Human Development Index and with the National Happiness Index. Their study is particularly convincing since it encompasses multiple measures of citizenship and it entails 105 countries, representing 95% of the world's population. All their measures of activism, freedom, and social participation document the value of adding value to society. The more people participate in social affairs and make a difference, the higher the chances that they will report higher levels of happiness. Lending further support to the findings of Perkins et al. in two empirical studies of Japanese citizens, Kobayashi (a) shows the strong links among citizenship, justice, and wellbeing.

In a study spanning 19 countries, Clench-Aas et al. show that high trust at the national level seems to buffer the negative effects of economic crises on personal satisfaction. Trust is a source of support and a compensatory factor. It can be considered a potent psychosocial good. The more citizens build social capital and trust their neighbors, the higher their life satisfaction (Di Martino and Prilleltensky, 2020; Di Martino et al., 2022). Mattering, we can argue, goes up with trust, since the latter signals to people that they are within their circle of care.

In a study in Portugal, Casanova et al. found that financial deprivation and psychosocial uncertainty tend to foment populist and extremist views. In a related finding, they report that access to financial resources protects against psychosocial uncertainty. This study reminds us that access to objective resources is crucial for the promotion of psychological safety and certainty. When psychosocial uncertainty is prevalent, people tend to endorse authoritarian leaders and policies that give them a sense of security. That is anathema to the common good. People seek to matter in exactly the wrong ways. Instead of trying to build community and solidarity, many citizens who live in uncertain regimes gravitate toward xenophobia (Prilleltensky and Prilleltensky, 2021).

Two studies show the role of injustice and discrimination in eroding a sense of mattering. Agueli et al. document the negative impact of exclusionary cultures on the wellbeing of LGBTQ citizens. Many of their daily experiences undermines their sense of belonging and mattering. Esposito et al. recount the vicissitudes of immigrants in detention centres, where their human rights are violated. Lack of fairness toward gender minorities and immigrants results in feelings of exclusion and diminished physical and mental health.

## The role of mattering in philosophical and cultural traditions

In an interesting comparative study, Bahl et al. explore psychological sense of community among young and old participants in Norway and India. It is worth noting that only the older Norwegians referred to macro-level systems as sources of help and support. The remaining three groups tended to focus more on the family as the place where people feel valued and add value. The authors remind us of the importance of going beyond the family to build the common good. There is a need to revive traditions where people feel valued and add value in the community, and not just at home.

Kobayashi (b) conducts a valuable analysis of diverse political philosophies and their conceptions of the common good. According to him, some philosophies, such as liberalism and libertarianism espouse individualistic conceptions of the common good, whereas communitarianism leans toward collective visions. Implied in the former is an individualistic notion of mattering. People can matter through their own efforts. In the latter, communitarian vision, people matter in connection to others. Most political philosophies contain visions of the good life and the good society, but they differ greatly on how to get there. Neoliberal policies, inspired by liberal thinkers, encourage citizens to be self-reliant and discourage governments from intervening in people's lives. Their implied motto is “you have the right to feel valued and be happy.” More communitarian societies are guided by a different motto: “we all have the right, and responsibility, to feel valued, and add value, so that we may all experience wellness and fairness.” We call the former “me cultures” and the latter “we cultures”. “We cultures” aspire to create a wellfair state, not a welfare state.

## Synthesis

Overall, the papers demonstrate that programs, practices, and policies aimed at making people feel that they matter contribute to both citizenship and wellbeing. When service recipients, adolescents, or people with various unique identities feel accepted and valued, their sense of belonging increases, and they are more likely to participate in civic affairs. Programs, practices, and policies have the capacity to humanize society by treating each individual with dignity and by creating social structures that guarantee access to basic necessities and afford opportunities for individuals, communities, and societies to thrive.

Nearly all contributors to this issue promote a move toward enhanced participation of service recipients and citizens in decisions affecting their lives. Authors assert that the co-creation of programs and policies is salutary at the personal and social levels. Co-creation enhances self-efficacy, competence, a growth mindset, and trust in oneself. These are all internal resources that translate into higher levels of wellbeing and higher chances of going beyond the self in the pursuit of meaning and mattering. It is telling that in the Perkins et al. article activism, freedom, and voter participation were all correlated with measures of national happiness.

Conversely, when social and economic conditions create uncertainty, or when inequality prevails, there are negative psychological as well as societal consequences. Uncertainty, for example, might push people to endorse extreme political views, usually because they promise people certainty and clarity. Inequality, in turn, makes people at the lower levels of the social hierarchy feel incompetent and devoid of agency. At the societal level, neoliberal policies erode traditions of solidarity, as may be seen in some Nordic countries, where trust has been one of the most precious national resources.

## Author contributions

IP, SD, and ON formulated a draft and revisions of the manuscript together. IP finalized the final version. SD and ON approved. All authors contributed to the article and approved the submitted version.

## Conflict of interest

The authors declare that the research was conducted in the absence of any commercial or financial relationships that could be construed as a potential conflict of interest.

## Publisher's note

All claims expressed in this article are solely those of the authors and do not necessarily represent those of their affiliated organizations, or those of the publisher, the editors and the reviewers. Any product that may be evaluated in this article, or claim that may be made by its manufacturer, is not guaranteed or endorsed by the publisher.
